# Association between prophylactic antibiotics for endometrial biopsy and the incidence of pelvic inflammatory disease: A retrospective cohort study

**DOI:** 10.1002/ijgo.16156

**Published:** 2025-01-16

**Authors:** Risa Ishida, Yusuke Sasabuchi, Kaori Koga, Gentaro Izumi, Daisuke Shigemi, Hiroki Matsui, Hideo Yasunaga, Yutaka Osuga

**Affiliations:** ^1^ Department of Obstetrics and Gynecology, Faculty of Medicine The University of Tokyo Tokyo Japan; ^2^ Department of Clinical Epidemiology and Health Economics, School of Public Health The University of Tokyo Tokyo Japan; ^3^ Department of Real‐World Evidence, Graduate School of Medicine The University of Tokyo Tokyo Japan; ^4^ Department of Obstetrics and Gynecology, Reproductive Medicine Chiba University Chiba Japan

**Keywords:** antibiotic prophylaxis, biopsy, early detection of cancer, endometrial neoplasms, pelvic inflammatory disease

## Abstract

Prophylactic antibiotics for endometrial biopsy may not reduce the risk of pelvic inflammatory disease, and their routine use may not be recommended.

Endometrial biopsies may cause complications, including pelvic inflammatory disease (PID) and tubo‐ovarian abscesses.^1^ However, the administration of prophylactic antibiotics during endometrial biopsy remains controversial. A Cochrane review indicated that there have been no completed randomized controlled trials of prophylactic antibiotics for endometrial biopsies.^2^ A small single‐center study reported that prophylactic antibiotics during cervical dilation and curettage in women with dysfunctional bleeding did not show a substantial decrease in subsequent PID.^3^

This retrospective cohort study was conducted using data from the Japan Medical Data Center database (JMDC Inc., Tokyo, Japan), which includes over 17 million de‐identified insurance claim records from corporate social insurance associations since 2005. We identified patients who underwent their first endometrial biopsy in an outpatient setting from 2005 to 2022. The exclusion criteria are detailed in Table S1. The exposure group included patients who received antibiotics (intravenous, oral, or vaginal) on the day of endometrial biopsy (the definition is provided in Table S1). The primary outcome was hospitalization with a diagnosis of PID within 30 days of the biopsy, with intravenous antibiotics administered on the day of admission (the definition is provided in Table S1). Secondary outcomes included the number of outpatient visits for PID, defined as visits requiring intravenous or oral antibiotics, and the length of hospital stay for PID. Continuous variables were compared using the Mann–Whitney *U* test, whereas categorical variables were compared using the chi‐squared or Fisher exact test. We used propensity score matching to compare the outcomes between the antibiotic and non‐antibiotic groups.^4^ Propensity scores were estimated using a logistic regression model with generalized estimating equations to account for clustering within hospitals. All the variables presented in Table S2 were included in the model. We then compared the outcomes between the groups in the propensity score‐matched cohort to estimate the effects of prophylactic antibiotics. A two‐sided significance level of *P* < 0.05 was set for all tests, and all statistical analyses were performed using Stata (version 18; StataCorp, College Station, Texas). This study was approved by the Institutional Review Board of the University of Tokyo, and the requirement for informed consent was waived owing to the de‐identified nature of the data.

Of 294 331 outpatients identified, 63 320 were assigned to the non‐antibiotic group and 15 830 to the antibiotic group, after 1:4 propensity score matching (Figure 1). Table S3 shows the baseline characteristics of the unmatched and propensity score‐matched cohorts. In the unmatched cohort, several differences were observed between the non‐antibiotic and antibiotic groups. Antibiotics were more commonly used in healthcare facilities with fewer beds, medical clinics, and very low‐volume hospitals than in their counterparts. After propensity score matching, all baseline characteristics were well‐balanced. Table 1 presents the outcomes of the unmatched and propensity score‐matched cohorts. In the matched cohort, numbers of hospitalizations for PID (62 [0.098%] and 12 [0.076%], respectively; *P* = 0.470) and outpatient visits for PID (329 [0.52%] and 71 [0.45%], *P* = 0.287) were not significantly different between the groups. The lengths of hospital stay for hospitalized patients were 8 days (interquartile range [IQR]: 3–29 days) in the non‐antibiotic group (*n* = 62) and nine days (IQR: 5–13 days) in the antibiotic group (*n* = 12), with no significant difference (*P* = 0.664).

**FIGURE 1:**
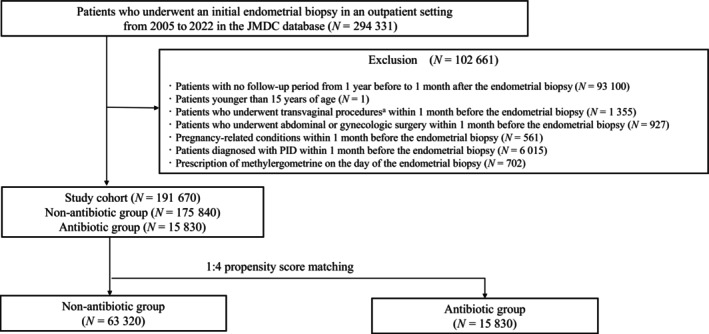
Study flow diagram showing the stratification and selection of patients. PID, pelvic inflammatory disease. ^a^Transvaginal procedures include miscarriage procedures, hysteroscopy, oocyte retrieval, embryo transfer, artificial insemination, hysterosalpingography, intrauterine device insertion, and intrauterine device removal.

**TABLE 1:** Outcomes before and after propensity score matching.

	Unmatched cohort			Propensity‐score matched cohort				
	Non‐antibiotic (*N* = 175 840)	Antibiotic (*N* = 15 830)	*P* value	Non‐antibiotic (*N* = 63 320)	Antibiotic (*N* = 15 830)	Risk difference	95% confidence interval	*P* value
Hospitalization for PID, *n* (%)	72 (0.041)	12 (0.076)	0.069	62 (0.098)	12 (0.076)	−0.00022	−0.0071–0.00027	0.470
Outpatient hospital visit for PID, *n* (%)	304 (0.17)	71 (0.45)	<0.001	329 (0.52)	71 (0.45)	−0.00071	−0.0019–0.00047	0.287

Abbreviation: PID, pelvic inflammatory disease.

There was no significant association between prophylactic antibiotic administration and subsequent hospitalizations, outpatient visits for PID, or length of hospital stay. These results suggest that prophylactic antibiotic administration may have limited effectiveness in preventing PID or its severity after endometrial biopsy. The main strength of our study was the use of a nationwide claims database. Additionally, our findings align with those of a previous study.^3^ The Japanese guidelines for obstetrics and gynecology do not mention the necessity of prophylactic antibiotics during endometrial biopsy.^5^ Reducing inappropriate antibiotic use can prevent the development of antimicrobial resistance, alleviate the financial burden on healthcare systems, and reduce the side effects associated with antibiotics. Therefore, it may be necessary to address the limited effectiveness of prophylactic antibiotics in the Japanese guidelines.

Here, antibiotics were more likely to be administered by smaller medical institutions rather than larger ones. Previous studies have shown that antibiotics are prescribed more frequently in clinics than in hospitals in Japan.^6^ This suggests potential differences in prescription practices among healthcare providers that should be highlighted when developing guidelines and government policies.

This study had several limitations. First, the database does not include clinical information such as abdominal pain, fever, or laboratory test results. Second, although we adjusted for confounding factors using propensity score matching, there may have been unmeasured confounding factors and residual bias. Third, this claims database includes employees of medium‐to‐large companies and their families, which precludes the generalizability of our findings to the general population. Lastly, we could not clearly differentiate between therapeutic and prophylactic antibiotics in the antibiotic group.

## AUTHOR CONTRIBUTIONS

All authors participated in the study design and data interpretation. H.M. and H.Y. managed the databases. R.I. and Y.S. analyzed the data and performed statistical analyses. R.I. drafted the first version of the manuscript. H.Y. and Y.O. contributed to the final version of the manuscript. The manuscript was finalized and approved by all the coauthors.

## FUNDING INFORMATION

This work was supported by grants from the Ministry of Health, Labor, and Welfare, Japan (grant no.: 23AA2003) and the Japan Agency for Medical Research and Development (grant no.: JP23gk0210033).

## CONFLICT OF INTEREST STATEMENT

HM was involved in a joint research project between Pfizer Inc. and the University of Tokyo (2021–2022). HM received a grant from JSPS KAKENHI (21H03159). The other authors declare no conflicts of interest for this article.

## Supporting information


Data S1.

## Data Availability

The data supporting the findings of this study are available from JMDC Inc. Restrictions apply to the availability of the data, which were used under a license for this study. The data can be accessed with permission from JMDC Inc. Requests for access to these datasets should be directed to https://www.jmdc.co.jp/en/inquiry/.

## References

[1] Gil Y , Capmas P , Tulandi T . Tubo‐ovarian abscess in postmenopausal women: a systematic review. J Gynecol Obstet Hum Reprod. 2020;49:101789. doi:10.1016/j.jogoh.2020.101789 32413520

[2] Thinkhamrop J , Laopaiboon M , Lumbiganon P . Prophylactic antibiotics for transcervical intrauterine procedures. Cochrane Database Syst Rev. 2013;31(5):CD005637. doi:10.1002/14651858.CD005637.pub3 17636811

[3] Al‐Ghoweri A , Jumaian N , Ababneh B , Ayoub SS . Efficacy of post‐dilation and curettage antibiotic prophylaxis in women with dysfunctional uterine bleeding in the prevention of pelvic inflammatory disease. JRMS. 2007;14:66‐68.

[4] Yasunaga H . Introduction to applied statistics—chapter 1 propensity score analysis. Ann Clin Epidemiol. 2020;2:33‐37. doi:10.37737/ace.2.2_33

[5] Japan Society of Obstetrics and Gynecology, Japan Association of Obstetricians and Gynecologists . Guidelines for Office Gynecology in Japan: Japan Society of Obstetrics and Gynecology and Japan Association of Obstetricians and Gynecologists. 2023 ed. Japanese Society of Obstetrics and Gynecology; 2023.

[6] AMR Clinical Reference Center . National antimicrobial use statistics. 2020 Accessed August 6, 2024. Available from: https://amrcrc.ncgm.go.jp/surveillance/010/1_NDB_stats_202010.pdf

