# Supplementary material for: Association between prophylactic antibiotics for endometrial biopsy and the incidence of pelvic inflammatory disease: A retrospective cohort study
Source: Int J Gynaecol Obstet. 2025 Jan 16;170(1):496–8. doi: 10.1002/ijgo.16156 (PMC12177263; doi:10.1002/ijgo.16156)
Supplement: Supplementary file 1 — Data S1. [file IJGO-170-496-s001.docx]

**Table S1. Exclusion Criteria and Definitions of Exclusion, Exposure, and Outcome.**

| Exclusion criteria | |
| --- | --- |
| Patients with no follow-up period from 1 year before to 1 month after the endometrial biopsy | |
| Patients younger than 15 years of age | |
| Patients who underwent transvaginal procedures (e.g., miscarriage procedures, hysteroscopy, oocyte retrieval, embryo transfer, artificial insemination, hysterosalpingography, insertion of intrauterine devices, or removal of intrauterine devices) within 1 month before the endometrial biopsy | |
| Patients who underwent abdominal or gynecological surgery within 1 month before the endometrial biopsy | |
| Patients with pregnancy-related conditions within 1 month before the endometrial biopsy | |
| Patients diagnosed with PID within 1 month before the endometrial biopsy | |
| Patients prescribed methylergometrine on the day of the endometrial biopsy | |
|  | Definition |
| Prophylactic antibiotics | WHO-ATC codes: J01, G01AA (Distinction between intravenous or oral was made by text search in Japanese) |
| Pelvic inflammatory disease | Acute salpingitis and oophoritis (ICD-10: N70.0); Salpingitis and oophoritis, unspecified (N70.9); Female pelvic inflammatory disease, unspecified (N73.9); Inflammatory disease of the uterus, unspecified (N71.9); Female pelvic peritonitis, unspecified (N73.5) |
| Pregnancy-related conditions | Pregnancy, childbirth, and the puerperium (ICD-10: O00–O9A) |
| Methylergometrine | WHO-ATC codes: G02AB01 |

WHO-ATC: World Health Organization-Anatomical Therapeutic Chemical, ICD-10: International Classification of Diseases, Tenth revision

**Table S2. Adjusted Variables in Propensity Score Matching.**

| Demographic | |
| --- | --- |
| Age | - |
| BMI (kg/m^2^) (five categories: ≤18.5, 18.5–24.9, 25.0–29.9, ≥30, and missing) | - |
| Smoking status (three categories: missing, smoker, and non-smoker) | - |
| Presence of spouse | - |
| Presence of children | - |
| Post-menopausal | Primary ovarian failure (E28.3), other ovarian dysfunction (E28.8), age-related osteoporosis with current pathological fracture (M80.0), age-related osteoporosis without current pathological fracture (M81.0), and menopausal and other perimenopausal disorders (N95) |
| Hospital visit frequency (four categories; very low: 0–25th percentile, low: 25–50th percentile, moderate: 50–75th percentile, and high: 75–100th percentile) | Classified hospital visits per patient in the past year into quartiles |
| Healthcare Facility Characteristic | |
| Number of beds (six categories: 0–19, 20–99, 100–199, 200–299, 300–499, and ≥500) | - |
| Type of healthcare facility (three categories: medical clinic, non-university hospital, and university hospital) | - |
| Hospital volume (four categories: very low: 0–25th percentile, low: 25–50th percentile, moderate: 50–75th percentile, and high: 75–100th percentile) | Hospital volume was calculated on the basis of the average annual number of endometrial biopsies performed per hospital |
| Medical Procedure on the Day of the Endometrial Biopsy | |
| Endometrial curettage | Original Japanese procedure codes |
| Urogenital or genital culture | Original Japanese procedure codes |
| Medical History in the last year | |
| Infertility | Female infertility associated with anovulation (ICD-10: N97) |
| Pregnancy-related condition | Pregnancy, childbirth, and the puerperium (ICD-10: O00–O9A) except for other disorders of the breast and disorders of lactation associated with pregnancy and the puerperium (O92) |
| Breastfeeding | Other disorders of the breast and disorders of lactation associated with pregnancy and the puerperium (ICD-10: O92) |
| Ovarian cyst | Benign neoplasm of ovary (ICD-10: D27) and noninflammatory disorders of the ovary, fallopian tube, and broad ligament (N83) |
| Pelvic inflammatory disease | Acute salpingitis and oophoritis (ICD-10: N70.0)  Salpingitis and oophoritis, unspecified (N70.9)  Female pelvic inflammatory disease, unspecified (N73.9)  Inflammatory disease of uterus, unspecified (N71.9)  Female pelvic peritonitis, unspecified (N73.5) |
| Diabetes mellitus | Diabetes mellitus (ICD-10: E10–E15) |
| Malignant neoplasms | Malignant neoplasms (ICD-10: C00–C96) |
| Collagen vascular disease | Seropositive rheumatoid arthritis (ICD-10: M05)  Other rheumatoid arthritis (M06)  Psoriatic and enteropathic arthropathies (M07)  Juvenile arthritis (M08)  Juvenile arthritis in diseases classified elsewhere (M09)  Systemic connective tissue disorders (M30–M36)  Ankylosing spondylitis (M45) |
| Immunodeficiency disorder | Human immunodeficiency virus disease (ICD-10: B20–B24)  Agranulocytosis (D70)  Functional disorders of polymorphonuclear neutrophils (D71)  Other disorders of white blood cells (D72)  Diseases of spleen (D73)  Other specified diseases with participation of lymphoreticular and reticulohistiocytic tissue (D76)  Immunodeficiency with predominantly antibody defects (D80)  Combined immunodeficiencies (D81)  Immunodeficiency associated with other major defects (D82)  Common variable immunodeficiency (D83)  Other immunodeficiencies (D84)  Transplanted organ and tissue status (Z94) |
| Uterine fibroid | Leiomyoma of uterus (D25) |
| Endometriosis | Endometriosis of the ovary (N80.1)  Endometriosis of the fallopian tube (N80.2)  Endometriosis of the pelvic peritoneum (N80.3)  Endometriosis of the rectovaginal septum and vagina (N80.4)  Endometriosis of the intestine (N80.5)  Endometriosis in the cutaneous scar (N80.6)  Endometriosis of the thorax (N80.8)  Endometriosis, unspecified (N80.9) |
| Adenomyosis | Endometriosis of the uterus (N80.0) |
| Chlamydial infection | Other sexually transmitted chlamydial diseases (A56)  Chlamydial conjunctivitis (A74) |
| Gonococcal infection | Gonococcal infection (A54) |
| Bacterial vaginosis | Other inflammation of the vagina and vulva (N760)  Additionally, we searched for bacterial vaginosis in Japanese text. |
| Sexually transmitted disease | Congenital syphilis　(A50)  Early syphilis　(A51)  Late syphilis　(A52)  Other and unspecified syphilis　(A53)  Trichomoniasis (A59)  Anogenital herpesviral [herpes simplex] infection (A60)  Other predominantly sexually transmitted diseases, not elsewhere classified　(A63)  Unspecified sexually transmitted disease (A64) |
| Vaginal candidiasis | Candidiasis of the vulva and vagina (B37.3) |
| Medical Procedure and Surgery in the Last Year | |
| Transvaginal procedure | Original Japanese procedure codes for hysteroscopy, oocyte retrieval, embryo transfer, artificial insemination, or hysterosonography |
| Insertion or removal of intrauterine devices | Original Japanese procedure codes |
| Abdominal surgery | Original Japanese procedure codes |
| Gynecological surgery | Original Japanese procedure codes |
| Medication in the Last Year | |
| Systemic corticosteroid | Prescriptions of WHO-ATC codes H02 totaling ≥30 days |
| Immunosuppressant | WHO-ATC code: L04 |
| Antineoplastic agent | WHO-ATC code: L01 |

BMI: body mass index, WHO-ATC: World Health Organization-Anatomical Therapeutic Chemical, ICD-10: International Classification of Diseases, Tenth revision

**Table S3. Study patients’ demographic characteristics.**

|  | Unmatched cohort | | | |  |  | Propensity score-matched cohort | | | |  |
| --- | --- | --- | --- | --- | --- | --- | --- | --- | --- | --- | --- |
|  | Non-antibiotic | | Antibiotic | | ASD |  | Non-antibiotic | | Antibiotic | | ASD |
|  | (N = 175,840) | | (N = 15,830) | |  |  | (N = 63,320) | | (N = 15,830) | |  |
|  | n | (%) | n | (%) |  |  | n | (%) | n | (%) |  |
| Demographic characteristic |  |  |  |  |  |  |  |  |  |  |  |
| Age (years, mean [IQR]) | 46.7 | 42-51 | 47.1 | 42-52 | 4.8 |  | 47.1 | 42-52 | 47.1 | 42-52 | 0.0 |
| Body mass index (kg/m^2^) |  |  |  |  |  |  |  |  |  |  |  |
| <18.50 | 16,565 | (9.4) | 1,541 | (9.7) | 1.1 |  | 6,100 | (9.6) | 1,541 | (9.7) | 0.3 |
| 18.50–24.99 | 88,931 | (50.6) | 8,113 | (51.3) | 1.4 |  | 32,900 | (52.0) | 8,113 | (51.3) | 1.4 |
| 25.00–29.99 | 17,813 | (10.1) | 1,532 | (9.7) | 1.5 |  | 5,888 | (9.3) | 1,532 | (9.7) | 1.3 |
| ≥30.00 | 5,894 | (3.4) | 441 | (2.8) | 3.3 |  | 1,850 | (2.9) | 441 | (2.8) | 0.8 |
| Missing | 46,637 | (26.5) | 4,203 | (26.6) | 0.1 |  | 16,582 | (26.2) | 4,203 | (26.6) | 0.8 |
| Smoking status |  |  |  |  |  |  |  |  |  |  |  |
| Smoker | 16,279 | (9.3) | 1,522 | (9.6) | 1.2 |  | 5,569 | (8.8) | 1,522 | (9.6) | 2.8 |
| Non-smoker | 126,216 | (71.8) | 11,238 | (71.0) | 1.7 |  | 45,656 | (72.1) | 11,238 | (71.0) | 2.5 |
| Missing | 33,345 | (19.0) | 3,070 | (19.4) | 1.1 |  | 12,095 | (19.1) | 3,070 | (19.4) | 0.7 |
| Presence of spouse | 109,446 | (62.2) | 9,595 | (60.6) | 3.3 |  | 37,984 | (60.0) | 9,595 | (60.6) | 1.3 |
| Presence of children | 89,273 | (50.8) | 7,723 | (48.8) | 4.0 |  | 30,788 | (48.6) | 7,723 | (48.8) | 0.3 |
| Post-menopausal | 1,459 | (0.8) | 172 | (1.1) | 2.6 |  | 547 | (0.9) | 172 | (1.1) | 2.3 |
| Hospital visit frequency |  |  |  |  |  |  |  |  |  |  |  |
| Very low: 0–25th percentile | 46,799 | (26.6) | 4,264 | (26.9) | 0.7 |  | 17,019 | (26.9) | 4,264 | (26.9) | 0.1 |
| Low: 25–50th percentile | 46,305 | (26.3) | 3,970 | (25.1) | 2.9 |  | 15,934 | (25.2) | 3,970 | (25.1) | 0.2 |
| Moderate: 50–75th percentile | 45,042 | (25.6) | 3,996 | (25.2) | 0.9 |  | 15,998 | (25.3) | 3,996 | (25.2) | 0.1 |
| High: 75–100th percentile | 37,694 | (21.4) | 3,600 | (22.7) | 3.1 |  | 14,369 | (22.7) | 3,600 | (22.7) | 0.1 |
| Healthcare facility characteristic | |  |  |  |  |  |  |  |  |  |  |
| Number of beds |  |  |  |  |  |  |  |  |  |  |  |
| 0–19 | 102,135 | (58.1) | 12,998 | (82.1) | 54.4 |  | 51,472 | (81.3) | 12,998 | (82.1) | 2.1 |
| 20–99 | 9,363 | (5.3) | 581 | (3.7) | 8.0 |  | 2,926 | (4.6) | 581 | (3.7) | 4.8 |
| 100–199 | 7,615 | (4.3) | 523 | (3.3) | 5.4 |  | 2,073 | (3.3) | 523 | (3.3) | 0.2 |
| 200–299 | 6,958 | (4.0) | 305 | (1.9) | 12.0 |  | 1,267 | (2.0) | 305 | (1.9) | 0.5 |
| 300–499 | 22,658 | (12.9) | 832 | (5.3) | 26.8 |  | 3,113 | (4.9) | 832 | (5.3) | 1.5 |
| ≥500 | 27,111 | (15.4) | 591 | (3.7) | 40.5 |  | 2,469 | (3.9) | 591 | (3.7) | 0.9 |
| Type of healthcare facility |  |  |  |  |  |  |  |  |  |  |  |
| Medical clinic | 102,135 | (58.1) | 12,998 | (82.1) | 54.4 |  | 51,472 | (81.3) | 12,998 | (82.1) | 2.1 |
| Non-university hospital | 63,774 | (36.3) | 2,398 | (15.1) | 49.8 |  | 10,657 | (16.8) | 2,398 | (15.1) | 4.6 |
| University hospital | 9,931 | (5.6) | 434 | (2.7) | 14.5 |  | 1,191 | (1.9) | 434 | (2.7) | 5.7 |
| Hospital volume |  |  |  |  |  |  |  |  |  |  |  |
| Very low: 0–25th percentile | 46,340 | (26.4) | 5,137 | (32.5) | 13.4 |  | 20,594 | (32.5) | 5,137 | (32.5) | 0.2 |
| Low: 25–50th percentile | 42,584 | (24.2) | 3,697 | (23.4) | 2.0 |  | 15,118 | (23.9) | 3,697 | (23.4) | 1.2 |
| Moderate: 50–75th percentile | 42,696 | (24.3) | 3,522 | (22.2) | 4.8 |  | 14,412 | (22.8) | 3,522 | (22.2) | 1.2 |
| High: 75–100th percentile | 44,220 | (25.1) | 3,474 | (21.9) | 7.6 |  | 13,196 | (20.8) | 3,474 | (21.9) | 2.7 |
| Medical procedure on the day of the endometrial biopsy |  | | | | |  |  |  |  |  |  |
| Endometrial curettage | 7 | (0.0) | 6 | (0.0) | 2.3 |  | 17 | (0.0) | 6 | (0.0) | 0.6 |
| Urogenital or genital culture | 9,456 | (5.4) | 3,779 | (23.9) | 54.2 |  | 15,416 | (24.3) | 3,779 | (23.9) | 1.1 |
| Medical history in the last year |  |  |  |  |  |  |  |  |  |  |  |
| Infertility | 6,272 | (3.6) | 673 | (4.3) | 3.5 |  | 2,569 | (4.1) | 673 | (4.3) | 1.0 |
| Pregnancy-related condition | 2,324 | (1.3) | 190 | (1.2) | 1.1 |  | 795 | (1.3) | 190 | (1.2) | 0.5 |
| Breastfeeding | 325 | (0.2) | 42 | (0.3) | 1.7 |  | 206 | (0.3) | 42 | (0.3) | 1.1 |
| Ovarian cyst | 30,187 | (17.2) | 2,409 | (15.2) | 5.3 |  | 9,135 | (14.4) | 2,409 | (15.2) | 2.2 |
| Pelvic inflammatory disease | 532 | (0.3) | 83 | (0.5) | 3.5 |  | 328 | (0.5) | 83 | (0.5) | 0.1 |
| Diabetes mellitus | 2,532 | (1.4) | 226 | (1.4) | 0.1 |  | 707 | (1.1) | 226 | (1.4) | 2.8 |
| Malignant neoplasms | 1,593 | (0.9) | 154 | (1.0) | 0.7 |  | 562 | (0.9) | 154 | (1.0) | 0.9 |
| Collagen vascular disease | 1,113 | (0.6) | 123 | (0.8) | 1.7 |  | 441 | (0.7) | 123 | (0.8) | 0.9 |
| Immunodeficiency disorder | 818 | (0.5) | 67 | (0.4) | 0.6 |  | 215 | (0.3) | 67 | (0.4) | 1.4 |
| Uterine fibroid | 14,574 | (8.3) | 1,411 | (8.9) | 2.2 |  | 5,212 | (8.2) | 1,411 | (8.9) | 2.4 |
| Endometriosis | 3,293 | (1.9) | 370 | (2.3) | 3.2 |  | 1,374 | (2.2) | 370 | (2.3) | 1.1 |
| Adenomyosis | 1,674 | (1.0) | 173 | (1.1) | 1.4 |  | 574 | (0.9) | 173 | (1.1) | 1.9 |
| Chlamydial infection | 2,031 | (1.2) | 396 | (2.5) | 10.1 |  | 1,645 | (2.6) | 396 | (2.5) | 0.6 |
| Gonococcal infection | 387 | (0.2) | 71 | (0.4) | 4.0 |  | 298 | (0.5) | 71 | (0.4) | 0.3 |
| Bacterial vaginosis | 1,513 | (0.9) | 874 | (5.5) | 26.8 |  | 3,368 | (5.3) | 874 | (5.5) | 0.9 |
| Sexually transmitted disease | 1,635 | (0.9) | 457 | (2.9) | 14.3 |  | 1,668 | (2.6) | 457 | (2.9) | 1.5 |
| Vaginal candidiasis | 8,639 | (4.9) | 1,255 | (7.9) | 12.3 |  | 5,060 | (8.0) | 1,255 | (7.9) | 0.2 |
| Medical procedures and surgeries in the last year |  | | | |  |  |  |  |  |  |  |
| Transvaginal procedure | 360 | (0.2) | 67 | (0.4) | 3.9 |  | 271 | (0.4) | 67 | (0.4) | 0.1 |
| Insertion or removal of an intrauterine device | 220 | (0.1) | 11 | (0.1) | 1.8 |  | 46 | (0.1) | 11 | (0.1) | 0.1 |
| Abdominal surgery | 1,756 | (1.0) | 148 | (0.9) | 0.7 |  | 487 | (0.8) | 148 | (0.9) | 1.8 |
| Gynecological surgery | 2,443 | (1.4) | 140 | (0.9) | 4.8 |  | 509 | (0.8) | 140 | (0.9) | 0.9 |
| Medication in the last year |  |  |  |  |  |  |  |  |  |  |  |
| Systemic corticosteroid | 3,585 | (2.0) | 296 | (1.9) | 1.2 |  | 985 | (1.6) | 296 | (1.9) | 2.4 |
| Immunosuppressant | 1,250 | (0.7) | 89 | (0.6) | 1.9 |  | 337 | (0.5) | 89 | (0.6) | 0.4 |
| Antineoplastic agent | 1,623 | (0.9) | 111 | (0.7) | 2.5 |  | 381 | (0.6) | 111 | (0.7) | 1.2 |

ASD: absolute standardized difference, IQR: interquartile range

An ASD <10% denotes a negligible difference between the groups.
